# Copper(I) Complexes of Mesoionic Carbene: Structural Characterization and Catalytic Hydrosilylation Reactions

**DOI:** 10.3390/molecules20047379

**Published:** 2015-04-22

**Authors:** Stephan Hohloch, Fenja Leena Duecker, Margarethe van der Meer, Biprajit Sarkar

**Affiliations:** Institut für Chemie und Biochemie, Anorganische Chemie, Freie Universität Berlin, Fabeckstraße 34-36, Berlin D-14195, Germany; E-Mails: Stephan.Hohloch@fu-berlin.de (S.H.); fenjaleena@zedat.fu-berlin.de (F.L.D.); margarethevandermeer@googlemail.com (M.M.)

**Keywords:** click chemistry, mesoionic carbenes, triazolylidenes, copper complexes, hydrosilylation, catalysis

## Abstract

Two series of different Cu(I)-complexes of “click” derived mesoionic carbenes are reported. Halide complexes of the type (MIC)CuI (with MIC = 1,4-(2,6-diisopropyl)-phenyl-3-methyl-1,2,3-triazol-5-ylidene (for **1b**), 1-benzyl-3-methyl-4-phenyl-1,2,3-triazol-5-ylidene (for **1c**)) and cationic complexes of the general formula [Cu(MIC)_2_]X (with MIC =1,4-dimesityl-3-methyl-1,2,3-triazol-5-ylidene, X = CuI_2_^−^ (for **2á**), 1,4-dimesityl-3-methyl-1,2,3-triazol-5-ylidene, X = BF_4_^−^ (for **2a**), 1,4-(2,6-diisopropyl)phenyl-3-methyl-1,2,3-triazol-5-ylidene, X = BF_4_^−^ (for **2b**), 1-benzyl-3-methyl-4-phenyl-1,2,3-triazol-5-ylidene, X = BF_4_^−^ (for **2c**)) have been prepared from CuI or [Cu(CH_3_CN)_4_](BF_4_) and the corresponding ligands, respectively. All complexes were characterized by elemental analysis and standard spectroscopic methods. Complexes **2á** and **1b** were studied by single-crystal X-ray diffraction analysis. Structural analysis revealed **2á** to adopt a cationic form as [Cu(MIC)_2_](CuI_2_) and comparison of the NMR spectra of **2á** and **2a** confirmed this conformation in solution. In contrast, after crystallization complex **1b** was found to adopt the desired neutral form. All complexes were tested for the reduction of cyclohexanone under hydrosilylation condition at elevated temperatures. These complexes were found to be efficient catalysts for this reaction. **2c** was also found to catalyze this reaction at room temperature. Mechanistic studies have been carried out as well.

## 1. Introduction

Carbonyl functions are commonly used groups in organic chemistry. Therefore, finding protocols to transform carbonyl or pseudocarbonyl functions into other functionalities, e.g., alcohols, is an essential goal [1,2]. Though, main group hydrides, e.g., those of boron or aluminum, have been found to perform this reaction, the lack for selectivity, functional group tolerance, as well as the need to use (over-) stoichiometric amounts of reagent renders them unattractive from both the practical and economic points of view. Additionally, for the reduction of sterically demanding substrates high amounts of a sacrificial reducing agent are often required (up to 40 equivalents) [3,4]. Although many transition metal catalysts have been developed for this reaction [5,6,7,8,9], many of these protocols still require the need for harsh conditions such as high pressures (if direct hydrogenations are applied) and/or high temperatures. Therefore hydrosilylations offer a good alternative since silanes have shown to be very efficient for this transformation. Furthermore, the use of silanes provides silyl-protected alcohols at first [10,11]. This saves possible reaction steps and is hence of high interest in organic chemistry.

Following the seminal work of Buchwald’s group from 2003 where they introduced a (NHC)CuCl complex (NHC = *N*-heterocyclic carbene) for the catalytic reduction of α,β-unsaturated enones [12], the Nolan group in particular has used NHC-based copper complexes as highly potent catalysts for the reductive transformation of carbonyls to silylethers [13,14,15,16,17,18]. In various reports they investigated factors influencing this transformation using neutral (NHC)CuX (X = I, Br, Cl) [13,14] or cationic [Cu(NHC)_2_]^+^ complexes [15,16]. Furthermore, comparable systems have also been proven to be able to reduce carbon dioxide catalytically to silylated formiate-esters [19]. Since copper(I) hydrides are known to perform this reaction [20,21,22,23,24,25], it is believed that the copper carbene mediated hydrosilylation also proceeds via a hydride intermediate. Such copper hydride intermediates have been isolated and studied independently by two different groups recently [19,26]. Surprisingly, all of the studies investigated the influences of the residues on the NHC-ligand or the counter ions used [17], while no protocols have been reported yet where the NHC ligand is replaced by another carbene donor, e.g., mesoionic carbenes (MIC). Only selected tertiary phosphine complexes have been reported in order to compared their catalytic activity to the carbene complexes [14].

A recent emerging class of carbenes are abnormal, or latterly called mesoionic carbenes, MIC [27,28,29,30,31,32,33,34,35]. One interesting subclass of these MICs are 1,2,3-triazol-5-ylidenes [36,37,38,39,40,41,42,43,44,45,46,47,48,49,50]. Since their first report in 2008 by the Albrecht group [36] these carbenes now belong to one of the “hot topics” in organometallic chemistry. In the field of catalysis, especially the carbene derivatives of 1,2,3,-triazoles, so-called triazolylidenes, have proven to show superior effects on the catalytic performances in several catalytic processes [36,37,38,39,40,41,42,43,44,45,46,47,48,49,50]. One reason for this is their easy accessibility, through the copper(I) catalyzed click [3+2] cycloaddition reaction between azides and alkynes [51,52]. Earlier, we have reported on the neutral coordination motif (MIC)CuI (**1**) [53] and the cationic motif of the general formula [Cu(MIC)_2_]^+^ (**2**) [54] and tested them as (pre)catalysts for the [3+2] cycloaddition between azides and alkynes. All of these complexes showed remarkable efficiencies and surpassed their NHC counterparts [53,55]. Additionally, we were able to prove the cationic mononuclear motif **2** to form more active (pre)catalysts than the neutral motif **1** does for the click reaction [53,54].

**Figure 1:**
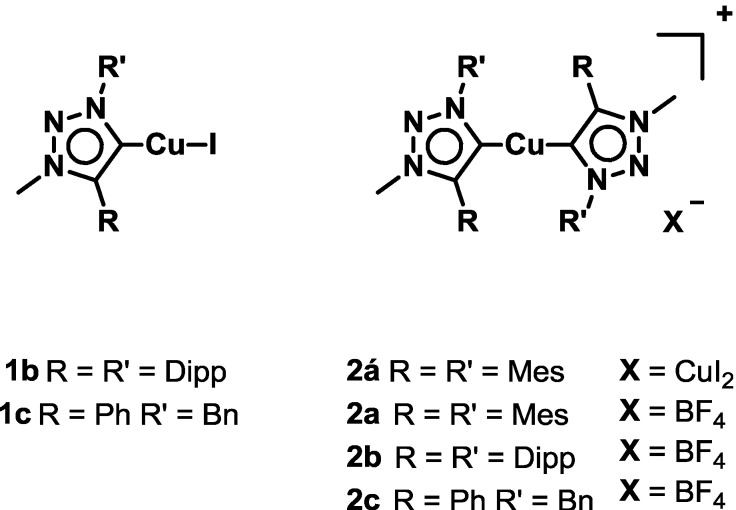
Overview of the copper complexes used.

In this contribution, we present the synthesis of various copper complexes with MIC ligands, namely the series **1b–c**, **2á**, **2a–c** and **3a–c** as shown in Figure 1.

Since comparable complexes have already shown superior activity for the “click reaction” [53,54,55], here the activity of these complexes towards the reduction of cyclohexanone under hydrosilylation conditions are investigated. Furthermore, for the most active complex, the conditions are optimized. Evidence from ^1^H-NMR spectroscopy is used to shed light on the mechanism of this transformation.

## 2. Results and Discussion

### 2.1. Synthesis and Characterization of Ligands and Complexes

**Figure 2:**
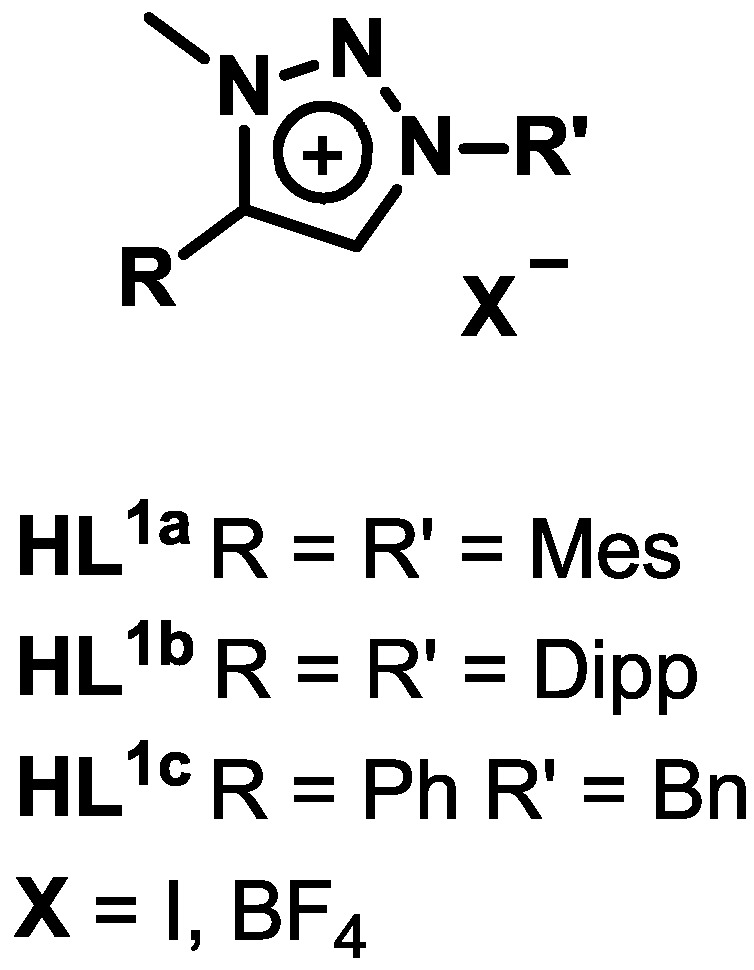
Ligands used in this work.

The triazolium salts **HL^1a^[I]** [56], **HL^1a^[BF_4_]** [27], **HL^1b^[BF_4_]** [57], **HL^1c^[I]** [58] and **HL^1c^[BF_4_]** [59] (Figure 2) were all synthesized following literature reports. In case of **HL^1b^[I]** the corresponding triazole [57] was methylated using iodomethane and the desired triazolium salt was obtained as a yellow solid in moderate yield. Proton NMR shift of the triazole-*5H* from 7.56 to δ 9.45 ppm and the occurrence of a singlet at δ 4.35 ppm corresponding to the *N*-methyl group clearly indicated the formation of the triazolium salt **HL^1b^[I]**.

Synthesis of the copper complexes was performed according to related literature known procedures [53,54,55].

**Scheme 1:**
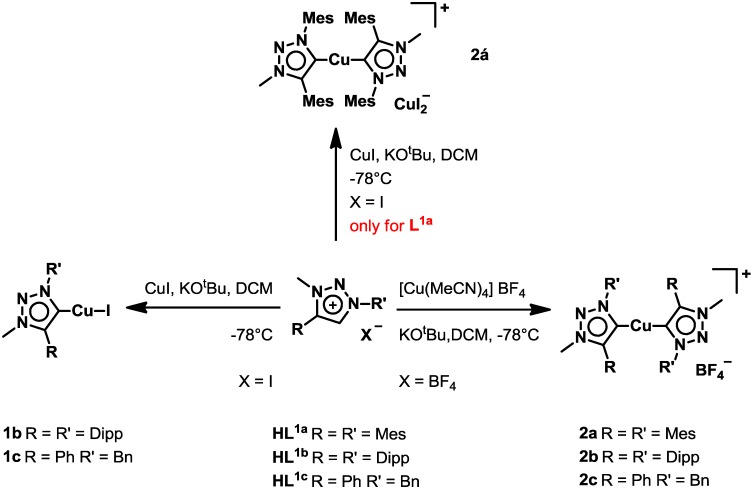
Synthesis of the mononuclear copper complexes **1b–c**, **2á** and **2a–c**.

In case of the halide complexes **1b** and **1c**, a direct metallation protocol using the triazolium salts **HL^1b^[I]** and **HL^1c^[I]**, potassium *tert*-butoxide and copper iodide at low temperatures in dichloromethane yielded the complexes in reasonable yield [53]. To our surprise, applying the same conditions using **HL^1a^[I]** we were not able to isolate the expected halide complex similar to motif **1b**/**1c** but rather the cationic complex **2á** with a [CuI_2_]^−^ counteranion was isolated (see discussion on crystal structures below). Using a similar protocol, starting from triazolium salts **HL^1a^[BF_4_]–HL^**1c**^[BF_4_]** and tetrakisacetonitrile copper(I) tetrafluoroborate as copper source, the cationic complexes **2a–c** are obtained [54]. (Scheme 1) Except for the complexes **1c** [53], **2á** [55] and **2c** [54], all other mononuclear complexes have not been reported in the literature to date. Elemental analysis proved the existence of all the desired complexes. Furthermore, disappearance of the triazole-*5H* was a first indication for the formation of a triazolylidene complex. ^13^C-NMR spectra of the complexes unambiguously proved the existence of copper carbene complexes, showing signals at δ 171.5 ppm for **1b** and signals at δ 166.3, 166.3, 166.8 ppm for **2á**, **2a** and **2b** respectively. Comparison of the proton NMR spectra of **2á** and **2a** showed that both compounds display the same spectra in solution for the complex cation, indicating, that these complexes adopt the same complex cation structure (see Figure S1). In contrast to that, NMR spectra of **1b** and **2b** differ significantly from each other proving these complex cations indeed exist as two different species (see Figure S2). Furthermore, the existence of all complexes was proven by high resolution mass spectrometry, showing the expected peaks for the corresponding compounds (see Experimental Section).

### 2.2. Crystal Structures 

**Figure 3:**
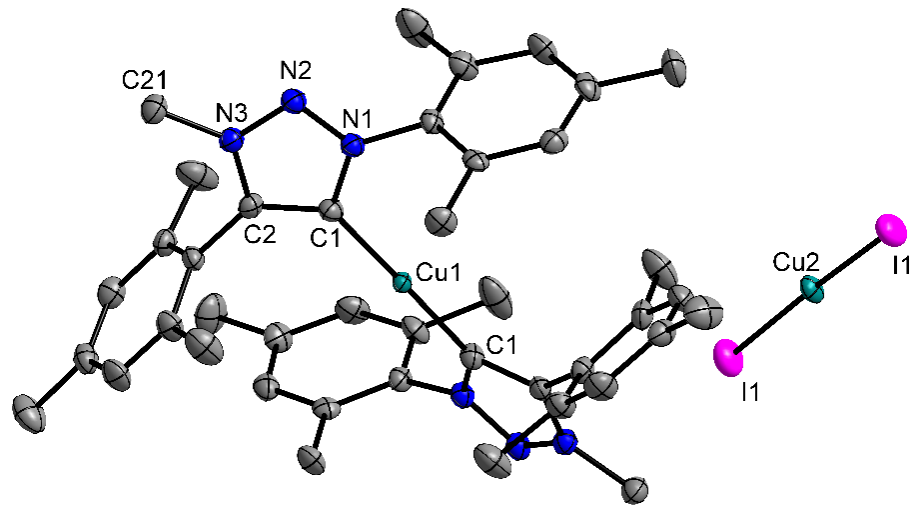
ORTEP-Plot of **2á**. Hydrogen atoms are omitted for clarity. Ellipsoids are shown at 50% probability level.

X-ray quality single crystals were obtained by slow diffusion at room temperature of *n*-hexane into a concentrated solution of dichloromethane in case of **2á**, and of *n*-hexane into THF solution in the case of **1b**. Attempts at growing single crystals of **1b** from dichloromethane solutions indicated that this complex likely activates the C-Cl bonds of dichloromethane and performs a Cl^−^ for I^−^ exchange at the Cu(I) center. Compound **2á** crystalizes in the monoclinic space group P2/c (Table 1), while **1b** was found to crystallize in the orthorhombic space group Pbca. In both molecules, the bonding situation in the triazolylidene ligand is best described as delocalized and the angle at the carbene center C1 is slightly smaller compared to free triazolium salts (see Table 2). Structural analysis of **2á** revealed that, as already indicated by ^1^H-NMR spectroscopy, the complex does not adopt the expected halide form, but instead shows the formation of a complex cation. The anion was determined to be a diiodocuprate anion [CuI_2_]^−^ (see Figure 3).

**Table 1:** Crystallographic information for complexes **2á** and **1b**.

	**2á**	**1b**
Chemical formula	C_42_H_50_N_6_Cu_1_ Cu_1_I_2_	C_27_H_37_N_3_Cu_1_I_1_
*M*_r_	1019.76	594.04
Crystal system, Space group	Monoclinic P2/c	orthorhombic Pbca
a (Å)	12.969(4)	16.3003(7)
b (Å)	8.262(2)	18.3924(8)
c (Å)	20.397(6)	18.5609(7)
α(°)	90	90
β (°)	94.489(7)	90
γ (°)	90	90
V (Å^3^)	2179(1)	5564.6(4)
Z	2	8
Densitiy (g cm^−3^)	1.554	1.418
F(000)	1016	2416
Radiation Type	MoK_α_	MoK_α_
μ (mm^−1^)	2.428	1.912
Crystal size	0.42 × 0.37 × 0.09	0.5 × 0.4 × 0.3
Meas. Refl.	22,753	71,218
Indep. Refl.	3869	7418
Obsvd. [ *I* > 2σ(*I*)] refl.	3000	6334
R_int_	0.0419	0.1543
R [F^2^ > 2σ(F^2^)], wR(F^2^), S	0.0376, 0.1086, 1.048	0.0448, 0.1276, 1.106
Δρ_max_, Δρ_min_ (e Å^−3^)	1.497, −0.607	1.970, −1.977
CCDC	965,501	

**Table 2:** Selected bond lengths (Å) and angles (°).

Atoms	**2á**	**1b**
Cu1-C1	1.877(5)	1.893(3)
Cu1-I1	-	2.394(1)
Cu2-I1	2.403(1)	-
C1-C2	1.379(7)	1.388(4)
C2-N3	1.367(6)	1.365(4)
N3-N2	1.325(6)	1.325(3)
N2-N1	1.334(5)	1.329(3)
N1-C1	1.371(6)	1.371(3)
C1-Cu1-C1	178.3(3)	-
C1-Cu1-I1	-	175.0(1)
I1-Cu2-I1	173.8(1)	-
N1-C1-C2	102.7(4)	102.0(2)
trz-trz	38.8(2)	-
N_trz_-R	78.7(2)	82.2(1)
C_trz_-R	84.4(2)	86.0(1)

Trz = triazolylidene.

The I1-Cu2-I1 angle is 173.84(5)°, showing an almost linear coordination of the Cu2 center in the anion.With a C1-Cu1-C1 angle of 178.3(3)°, the copper center Cu1 is linearly coordinated by thethe two MIC ligands. The Cu1-C1 distance is 1.877(5) Å and is therefore in the expected range as compared to similar copper carbene complexes [53,54,55]. The triazolylidene units are tilted by 38.8(2)°. The mesityl substituents are almost perpendicular to the triazolylidene ring plane displaying dihedral angles of 78.7(2)° and 84.4(2)° respectively (see Figure 3 and Table 2).

In a recent contribution, we have formulated complex **2á** to adopt the form [Cu(MIC)I] [55]. This formulation was done based on NMR and elemental analysis data. As can be seen from Scheme 1, **2á** and the corresponding [Cu(MIC)I] would deliver exactly identical C, H, N values in elemental analysis. It is only through the synthesis of the new complex **2a**, and the comparison of its NMR data with **2á**, that we have now been able to make the correct formulation. The results from single crystal X-ray diffraction confirms the formulation of **2á** as containing a complex cation. Thus, it is seen that because of the ability of Cu(I) to adopt many different kinds of coordination environments, it is sometimes impossible to predict the structure of such complexes without the help of single crystal X-ray diffraction data.

**Figure 4:**
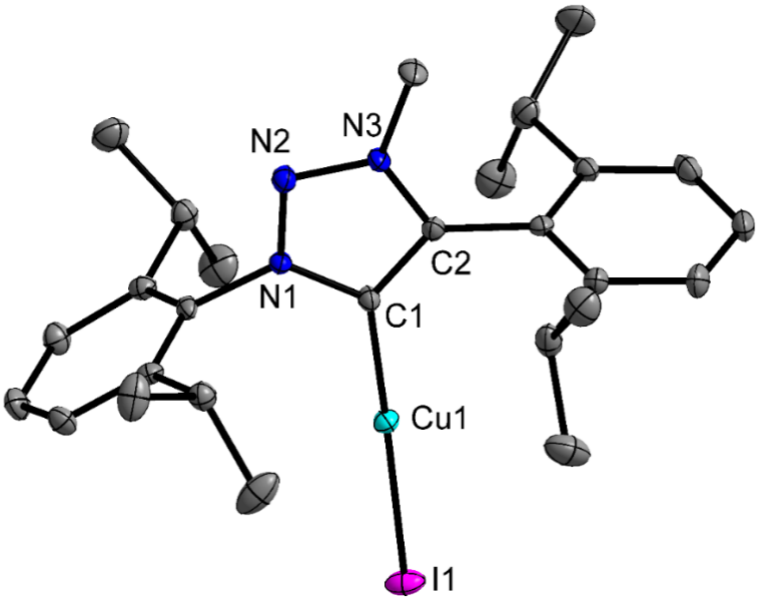
ORTEP-Plot of the complexes **1b**. Hydrogen atoms are omitted for clarity. Ellipsoids are shown at 50% probability.

For complex **1b** (Figure 4) the C1-Cu1 distance was found to be 1.882(1) Å fitting in the range of previously reported copper triazolylidene complexes [53,54,55]. Additionally, the Cu1-I1 bonds lengths is in the expected region with 2.394(1) Å. With a C1-Cu1-I1 angle of 175.0(1)° the copper atom is best described as linear coordinated. The diisopropyphenyl residues are almost perpendicular to the plane of the triazolylidene ring displaying dihedral angles of 82.2(1)° and 86.0(1)° respectively.

The *C*-substituent of the triazolylidene ligand in the complexes is much more tilted towards the triazolylidene ring in contrast to the *N*-substituent. This is most likely due to steric repulsion of the *C*-substituent and the methyl group on N3.

### 2.3. Catalysis

Copper(I)-complexes with NHC ligands have proven to be potent catalysts for various transformations. Especially the [3+2] cycloaddition reaction between azides and alkynes (CuAAC) [60,61,62] and the reductive hydrosilylation of ketones using alkyl-silanes were found to be well catalyzed by these complexes [13,14,15,16,17,18]. In recent years our group has made some efforts to prove, that complexes of the more electron donating 1,2,3-triazolylidenes form more active catalysts than NHC complexes do [55]. Concerning copper, we [53,54,55] and others [57] were already able to prove that 1,2,3-triazolylidene derived copper complexes form extremely potent catalyst for various trasformations [53,54,55,57,58]. Thus, we were now interested in the catalytic activity of these complexes in the hydrosilylation reaction. For this purpose we synthesized the nine complexes (*vide infra*). Within these complexes we varied the steric properties of the ligands, to determine first the coordination mode and secondly the steric properties needed to build active catalysts for this transformation.

**Scheme 2:**
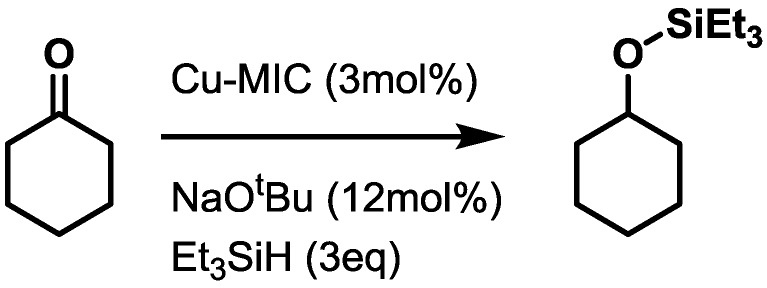
Reduction of cyclohexane using a copper-MIC catalyst and triethylsilane.

We have selected the reduction of cyclohexanone to cyclohexyltriethylsilylether using triethylsilane with sodium *tert.*-butoxide in degassed THF solutions as a test reaction. (Scheme 2) The results are shown in Table 3.

**Table 3:** Comparison of the catalytic activity of the complexes **1b**–c, **2á** and **2a–c** for the test reaction shown in Scheme 2 ^a^.

Catalyst	Conversion [%]
**2á**	>99
**1b**	91
**1c**	97
**2a**	>99
**2b**	99
**2c**	>99

^a^*Reagents and conditions*: Cyclohexanone (0.5 mmol), NaO*t*-Bu (12 mol %), Catalyst (3 mol %), 70 °C, degassed THF (2 mL) overnight. Conversions were determined via GC-MS analysis using hexadecane as an internal standard.

As can be observed from Table 3, all complexes form very active catalysts for this transformation. However, regarding the conversions it seems like, that the cationic dicarbene complexes **2á** and **2a–c** form the slightly more active catalysts compared to the neutral complexes **1b** and **1c**. As expected from structural analogy, complexes **2á** and **2a** display the same activity. Blind catalytic runs under the conditions shown in Table 3 without any Cu-source and with CuI or [Cu(CH_3_CN)_4_]BF_4_ did not deliver any product.

**Figure 5:**
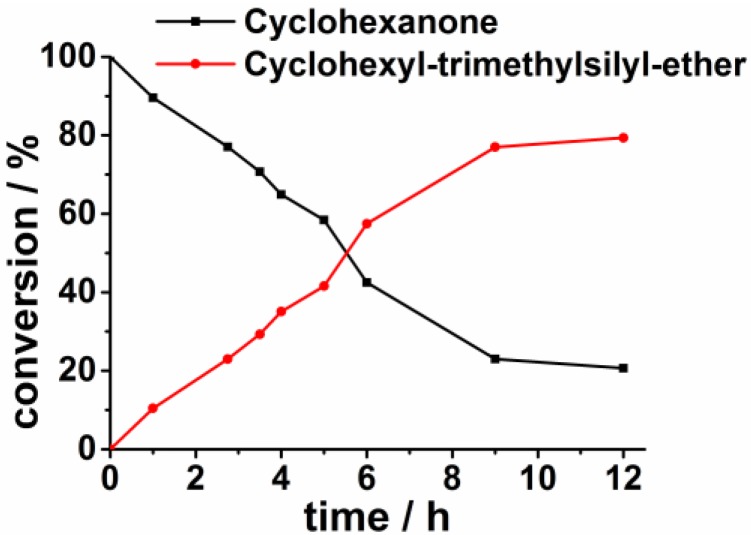
Conversion *versus* time plot using complex **2á** as a catalyst.

After establishing that the cationic coordination motif 2 formed the most active catalysts we were now interested in the influence of the reaction temperature. Performing the reaction at room temperature, only complex **2c** still provides good yield (92% conversion), while the complexes **2a** and **2b** are both inactive at this temperature (<10% conversion at room temperature). Raising the reaction temperature to 40 °C for **2a** and **2b** resulted in only small conversions (<10%). At 60 °C **2a** displayed a conversion of more than 95%. Thus, from the temperature-reaction profile it can be concluded, that in case of sterically demanding residues on the triazolylidene ligand, the reaction needs to overcome an activation barrier to proceed. The temperature required to overcome this barrier is somewhere in between 40 to 60 °C. Furthermore, we have performed time-dependence of catalytic conversions using catalyst **2á** to show that the reaction needs to run at least 12 h to obtain good results. The reaction conditions for the time-dependence were identical to those shown in Table 3, except that 10 mL THF was used instead of 2 mL. The slightly smaller conversions from the time dependence compared to Table 3 results from the fact, that the time dependent reaction was carried with more amount of solvent (see Table 3 and Figure 5).

**Scheme 3:**
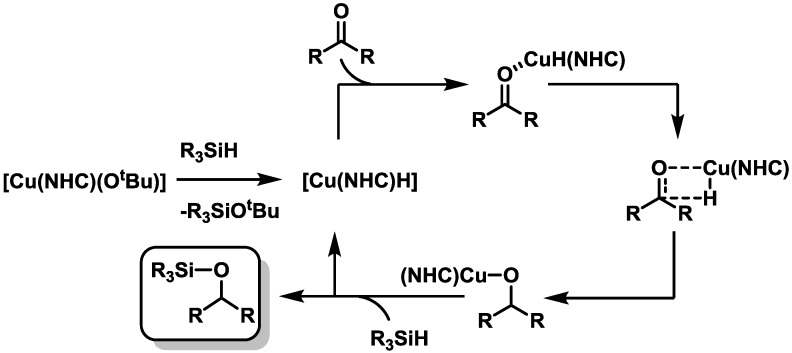
Proposed reaction mechanism for the copper catalyzed hydrosilylation using silanes as hydrogen source. Adapted from ref. [19].

Finally, we were interested in mechanistic details of this reaction. In the literature there are several reports that the active species in this formation is a copper hydride species. Hou and co-workers were already able to identify a copper hydride species* in situ*, which is formed by combining the (NHC)Cu-O^t^Bu complex with silane [19]. The silane is believed to undergo a σ-bond metathesis reaction with the copper alkoxide to form a siloxide species and the copper hydride. The driving force of this reaction would be the formation of a stable silicon oxygen bond. The copper hydride will then undergo a second σ-bond metathesis with the carbonyl to recreate a copper alkoxide species that can then be silylated by a third σ-bond metathesis to regenerate the copper hydride and form the desired silyl ether (see Scheme 3) [13,26].

Inspired by this, we wanted to see if we could identify a hydride complex by ^1^H-NMR spectroscopy. Since the complexes of **L^1a^** and **L^1b^** display signals themselves in the expected regions for a copper hydride species, we decided to investigate this reaction using complex **1c**. Since we start from a halogen containing complex we decided not to use an external base (alkoxide) but start directly from the halide complex, hoping that the formation of a silicon iodine bond would be enough driving force to generate the desired hydride complex. Indeed, mixing complex **1c** together with an excess of triethylsilane in dried deuterated THF and subsequent heating for one hour, resulted in the formation of a small signal at δ 2.38 ppm in the ^1^H-NMR spectrum pointing towards the formation of a copper hydride species (see Figure S3). Hou* et al.* reported the hydride signal at a chemical shift of δ 2.60 ppm in benzene-d_6_[19]. These findings point out that the mechanism for the copper triazolylidene catalyzed hydrosilylation of ketones follows the same pathway as the copper NHC catalyzed version of this reaction does. The catalytic hydrosilyation reactions presented here require higher temperatures and times to produced comparable conversions as compare to Cu-NHC complexes [13,14,15,16,17,18]. However, it remains to be seen if further optimization of reaction conditions might actually make the catalysts presented here better than their Cu-NHC counterparts. Such an observation has already been made for click-catalysis [53,54,55].

## 3. Experimental Section

### 3.1. General Information

All the reagents were used as supplied. The solvents used for metal complex synthesis were dried and distilled under argon and degassed by common techniques prior to use. ^1^H- and ^13^C{^1^H}-NMR spectra were recorded on a Jeol ECS 400 spectrometer (Jeol, Munich, Germany). Elemental analyses were performed by the Perkin-Elmer Analyzer 240 (Perkin-Elmer, Rodgau, Germany) and a Elementar Vario EL III. Mass spectrometry was performed on an Agilent 6210 ESI-TOF (Agilent, Waldbronn, Germany). GC-MS analysis was performed on a Varian Saturn 2100C linked (Varian, Darmstadt, Germany), Column: Varian factory four capillary column VF-5ms, Method: 50 °C to 250 °C heating rate 20 K/min).

### 3.2. X-ray Crystallography

Single crystals of **2á** and **1b** suitable for X-ray diffraction were obtained by layering concentrated solutions of the corresponding complexes in dichloromethane or THF with *n*-hexane at room temperature. X-ray structural studies were performed on a Bruker Smart AXS diffractometer. Data were collected at 100(2) K using graphite-monochromated Mo Kα radiation (*λ*_α_ = 0.71073 Å). The strategy for the data collection was evaluated by using the Smart software. The data were collected by the standard “omega-scan” techniques, and were scaled and reduced using the Saint+ software. The structures were solved by direct methods using SHELXS-97 and refined by full matrix least-squares with SHELXL-97, refining on *F^2^* [63]. CCDC 965501 and 1009488 contain the CIF-Files for **2á** and **1b** respectively. These data can be obtained free of charge from ref [64]. 

### 3.3. Synthesis of Triazolium Salts

The compounds **HL^1a^[I]** [56], **HL^1a^[BF_4_]** [27] ,**HL^1b^[BF_4_]** [57], **HL^1c^[I]** [58] and **HL^1c^[BF_4_]** [59] were synthesized according to the literature.

*1,4-Di(2,6-diisopropylphenyl)-3-methyl-H-1,2,3-triazolium iodide* (**HL^1b^[I]**). 1,4-Bis(2,6-diisopropylphenyl)-*H*-1,2,3-triazole [57] (1 equiv, 250 mg, 0.65 mmol) was refluxed in acetonitrile (25 mL) with methyl iodide (20 equiv. excess) at 60 °C overnight. The solvent was evaporated and the solid residue was dissolved in DCM. After addition of hexane the precipitate was filtered to yield the product as a yellow solid in a yield of 190 mg (0.36 mmol, 55%). ^1^H-NMR (400 MHz, CDCl_3_; 25 °C, TMS): δ = 9.45 (s, 1H, triazole-H), 7.67–7.61 (m, 2H, *p*-aryl-H), 7.41–7.38 (m, 4H, *m*-aryl-H), 4.35 (s, 3H, N-CH_3_), 2.38–2.30 (m, 4H, *i*Pr-CH), 1.34–1.18 ppm (m, 24H, *i*Pr-CH_3_); ^13^C{^1^H}-NMR (100 MHz, CDCl_3_; 25 °C, TMS): δ = 148.9 (triazole-C), 145.1, 142.0, 133.5, 133.5, 133.3, 130.7, 124.9, 124.4, 117.2 (all aryl-C), 40.3 (N-CH_3_), 32.1, 29.3 (*i*Pr-CH), 25.2, 24.5, 23.9, 23.2 ppm (all *i*Pr-CH_3_). HRMS (ESI): *m/z* = calculated for [C_27_H_38_N_3_^+^] 404.3066; found 404.3041. CHN analysis: C_27_H_38_IN_3_·0.1CH_2_Cl_2_: calculated C 60.19, H 7.12, N 7.77; found C 59.98, H 7.847, N 7.798.

#### 3.3.1. Synthesis of Copper(I)-Carbene Complexes

The complexes, **1c** [53], **2c** [54] were obtained through known synthetic routes.

*Bis(1,4-dimesityl-3-methyl-1,2,3-triazol-5-ylidene)copper(I) diiodocuprate* (**2á**). **HL^1a^[I]** (1 equiv, 89 mg, 0.2 mmol) and copper(I) iodide (2 equiv, 76 mg, 0.4 mmol) were dissolved in dried and degased DCM (10 mL) under inert gas atmosphere and cooled to −78 °C with an acetone/dry-ice mixture under stirring. KO*t*-Bu (3 equiv, 67 mg, 0.6 mmol) was suspended in DCM (5 mL) in a separate Schlenk tube and then slowly added to the reaction mixture. The solution was slowly warmed to room temperature while stirring overnight. The precipitated solid was filtrated and the volatiles were reduced to about 2 mL under high vacuum. To precipitate the product, hexane was added to the solution and then cooled in the fridge for several hours. The product was obtained as a white solid in a yield of 75% (76 mg, 0.15 mmol). ^1^H-NMR (400 MHz, CD_2_Cl_2_; 25 °C, TMS): δ = 6.93 (s, 2H, mesityl-H), 6.85 (s, 2H, mesityl-H), 3.77 (s, 3H, N-CH_3_), 2.41–2.40 (m, 6H, *p*-CH_3_), 1.74–1.70 ppm (m, 12H, *o*-CH_3_); ^13^C{^1^H}-NMR (100 MHz, CD_2_Cl_2_; 25 °C, TMS): δ = 166.3 (carbene-C), 147.3, 140.5, 140.3, 137.7, 135.9, 133.7, 129.1, 128.8, 122.5 (allaryl-C), 36.4 (N-CH_3_), 21.1, 21.1 (both *o*-mesityl-CH_3_), 19.7, 16.8 ppm (both *p*-mesityl-CH_3_). HRMS (ESI): *m/z* = calculated for [C_42_H_50_CuN_6_^+^] 701.3393; found 701.3367.elemental analysis: C_42_H_50_Cu_2_I_2_N_6_·1.5 hexane: calculated C 53.31, H 6.23, N 7.31; found C 53.70, H 6.67, N 8.08.

*(Iodido){1,4-di(2,6-diisopropylphenyl)-3-methyl-1,2,3-triazol-5-ylidene}copper(I)* (**1b**). **HL^1b^[I]** (1 equiv, 106 mg, 0.2 mmol) and copper iodide (2 equiv, 76 mg, 0.4 mmol) were dissolved in DCM (10 mL) and the solution was cooled to −78 °C. KO*t-*Bu (3 equiv, 67 mg, 0.0006 mol) was suspended in DCM (5 mL) in a separate Schlenk tube and slowly added to the reaction mixture. After work-up similar to **2á**, the product was obtained as a white solid in a yield of 67% (78 mg, 0.134 mmol). ^1^H-NMR (400 MHz, CD_2_Cl_2_; 25 °C, TMS): δ = 7.59–7.53 (m, 2H, *p*-aryl-H), 7.35–7.33 (m, 4H, *m*-aryl-H), 3.86 (s, 3H, N-CH_3_), 2.44–2.31 (m, 4H, *i*Pr-CH), 1.29–1.18 ppm (m, 24H, *i*Pr-CH_3_); ^13^C{^1^H}-NMR (100 MHz, CD_2_Cl_2_; 25 °C, TMS): δ = 171.5 (carbene-C), 148.9, 145.9, 145.0, 135.7, 131.5, 131.3, 124.1, 123.7, 123.1 (alle Aryl-C), 36.7 (N-CH_3_), 31.6, 28.9 (both *i*Pr-CH), 25.0, 24.3, 23.5, 22.8 ppm (all *i*Pr-CH_3_). HRMS (ESI): *m/z* = calculated for [C_54_H_74_CuN_6_^+^] 869.5271; found 869.5257. elemental analysis: C_27_H_37_N_3_CuI·0.2 CH_2_Cl_2_: calcd. C 53.19, H 6.14, N 6.83; found C 53.36, H 6.95, N 6.56.

*Bis(1,4-dimesityl-3-methyl-1,2,3-triazol-5-ylidene)copper(I) tetrafluoroborate* (**2a**). **HL^1a^[BF_4_]** (2 equiv, 81 mg, 0.2 mmol) and tetrakis(acetonitrile)copper(I) tetrafluoroborate (1 equiv, 31 mg, 0.1 mmol) were dissolved in DCM (10 mL) and cooled to −78 °C. KO*t*-Bu (6 equiv, 67 mg, 0.6 mmol) was suspended in DCM (5 mL) in a separate Schlenk tube and slowly added to the reaction mixture. After work-up as described for **2á** the product was obtained as a white solid in a yield of 71% (56 mg, 0.071 mmol). ^1^H-NMR (400 MHz, CD_2_Cl_2_; 25 °C, TMS): δ = 6.95 (s, 4 H, mesityl-H), 6.85 (s, 4 H, mesityl-H), 3.76 (s, 6H, N-CH_3_), 2.41 (s, 6H, *p*-CH_3_), 2.40 (s, 6H, *p*-CH_3_), 1.74 (s, 12H, *o*-CH_3_),1.70 ppm (s, 12H, *o*-CH_3_); ^13^C{^1^H}-NMR (100 MHz, CD_2_Cl_2_; 25 °C, TMS): δ = 166.3 (carbene-C), 147.3, 140.5, 140.3, 135.9, 133.7, 129.1, 128.8, 122.5 (all aryl-C), 36.2 (N-CH_3_), 21.1, 21.0 (both *o*-mesityl-CH_3_), 19.7, 16.8 ppm (both *p*-mesityl-CH_3_). HRMS (ESI): *m/z* = calculated for [C_42_H_50_CuN_6_^+^] 701.3393; found 701.3391. elemental analysis for C_42_H_50_BCuF_4_N_6_·0.66CH_2_Cl_2_: calcd. C 60.58, H 6.12, N 9.94; found C 60.87, H 6.71, N 9.67.

*Bis(1,4-di(2,6-diisopropylphenyl)-3-methyl-1,2,3-triazole-5-ylidene)copper(I) tetrafluoroborate* (**2b**). Synthesized in the same way as **2a** starting from **HL^1b^[BF_4_]** (98.3 mg, 0.0002 mol), [Cu(MeCN)_4_]BF_4_ (31.4 mg, 0.0001 mol) and KO*t*-Bu (6 equiv, 67 mg, 0.6 mmol) The product was obtained as a red dish-white solid in a yield of 54% (52 mg, 0.054 mmol). ^1^H-NMR (400 MHz, CD_2_Cl_2_; 25 °C, TMS): δ = 7.51–7.47 (m, 4H, *p*-aryl-H), 7.18–7.14 (m, 8H, *m*-aryl-H), 3.65 (s, 6H, N-CH_3_), 2.13–2.05 (m, 8H, *i*Pr-CH), 1.06–1.04 (d, 12H, *i*Pr-CH_3_, *J* = 8 Hz), 0.99–0.98 (d, 12H, *i*Pr-CH_3_, *J* = 4 Hz), 0.77–0.75 (d, 12H, *i*Pr-CH_3_, *J* = 8 Hz), 0.68–0.66 ppm (d, 12H, *i*Pr-CH_3_, *J* = 8 Hz); ^13^C{^1^H}-NMR (100 MHz, CD_2_Cl_2_; 25 °C, TMS): δ = 166.8 (carbene-C), 148.4, 146.8, 144.7, 135.8, 131.7, 131.5, 124.1, 124.1, 123.7, 123.0 (all aryl-C), 36.7 (N-CH_3_), 31.3, 28.7 (both *i*Pr-CH), 24.5, 23.7, 23.6, 22.6 ppm (all *i*Pr-CH_3_). HRMS (ESI): *m/z* = calculated for [C_54_H_74_CuN_6_^+^] 869.5271; found 869.5266. elemental analysis for C_54_H_74_N_6_Cu_1_B_1_F_4_ 0.5 CH_2_Cl_2_ calcd. C 65.46 H 7.56 N 8.40, found C 65.13 H 7.90 N 8.78.

#### 3.3.2. Hydrosilylations of Cyclohexanone

The respective copper(I)-complex (3 mol %) and sodium *tert*-butoxide (6 mg, 0.06 mmol, 12 mol %) were dissolved in dried and degased THF (2 mL) under inert gas atmosphere. After stirring for several minutes triethylsilane (174 mg, 1.5 mmol, 3 equiv.) and cyclohexanone (44 mg, 0.5 mmol, 1 equiv.) were added successively. The reaction mixture was stirred overnight at the corresponding temperature (rt, 40 °C, 60 °C, 70 °C). Afterwards the conversions were determined via GC-MS analysis using hexadecane as an internal standard.

## 4. Conclusions 

We have presented the synthesis and characterization of six copper MIC complexes. Two neutral complexes **1b–c** of the type[(MIC)CuI], four cationic complexes **2á** and **2a–c** of the cationic type described by the formula [(MIC)_2_Cu]^+^.All these complexes were characterized by standard spectroscopic methods including multi nuclear NMR spectroscopy, elemental analysis, single crystal X-ray diffraction analysis and mass spectrometry. In contrast to our previous formulations [55], X-ray diffraction analysis revealed that complex **2á** does not adopt the expected neutral form [(MIC)CuI], but instead adopts a cationic form, displaying the same cationic coordination motif as **2a** does. Comparison of NMR data of **2á** and **2a** affirmed this conformation to exist in solutionas well. Attempts at growing crystals of **1b**, from dichloromethane, showed that the complex most likely performs activation of dichloromethane whereby halogen exchange from iodine to chlorine occurs. However, we were able to obtain suitable crystals of **1b** from a THF-hexane mixture. All mononuclear complexes displayed high activity in thecopper catalyzed hydrosilylation of cyclohexanone. The cationic complexes **2á** and **2a–c** were proven to form the best catalysts amongst the ones discussed here. While **2a** and **2b** are only active at elevated temperatures, for **2c** reactivity is already induced at room temperature. We have performed time dependent catalysis proving that the conversion needs at least to proceed 10 h to achieve high yields. Mechanistic studies revealed in accordance to NHC copper complexes that the initiation step of the catalytic cycle is the formation of a copper hydride species as has been proven by ^1^H-NMR spectroscopy. This proves that the reaction mechanism for NHC complexes and for MIC complexes is likely similar. Further investigations, regarding especially the substrate scope and CO_2_ reduction, but also the activation of dichloromethane by these complexes are currently being performed in our laboratories.

## Supplementary Materials

Supplementary materials can be accessed at: http://www.mdpi.com/1420-3049/20/04/7379/s1.
