# Supplementary material for: Copper(I) Complexes of Mesoionic Carbene: Structural Characterization and Catalytic Hydrosilylation Reactions
Source: Molecules. 2015 Apr 22;20(4):7379–95. doi: 10.3390/molecules20047379 (PMC6272443; doi:10.3390/molecules20047379)
Supplement: Supplementary file 1 [file molecules-20-07379-s001.pdf]

Supplementary Materials

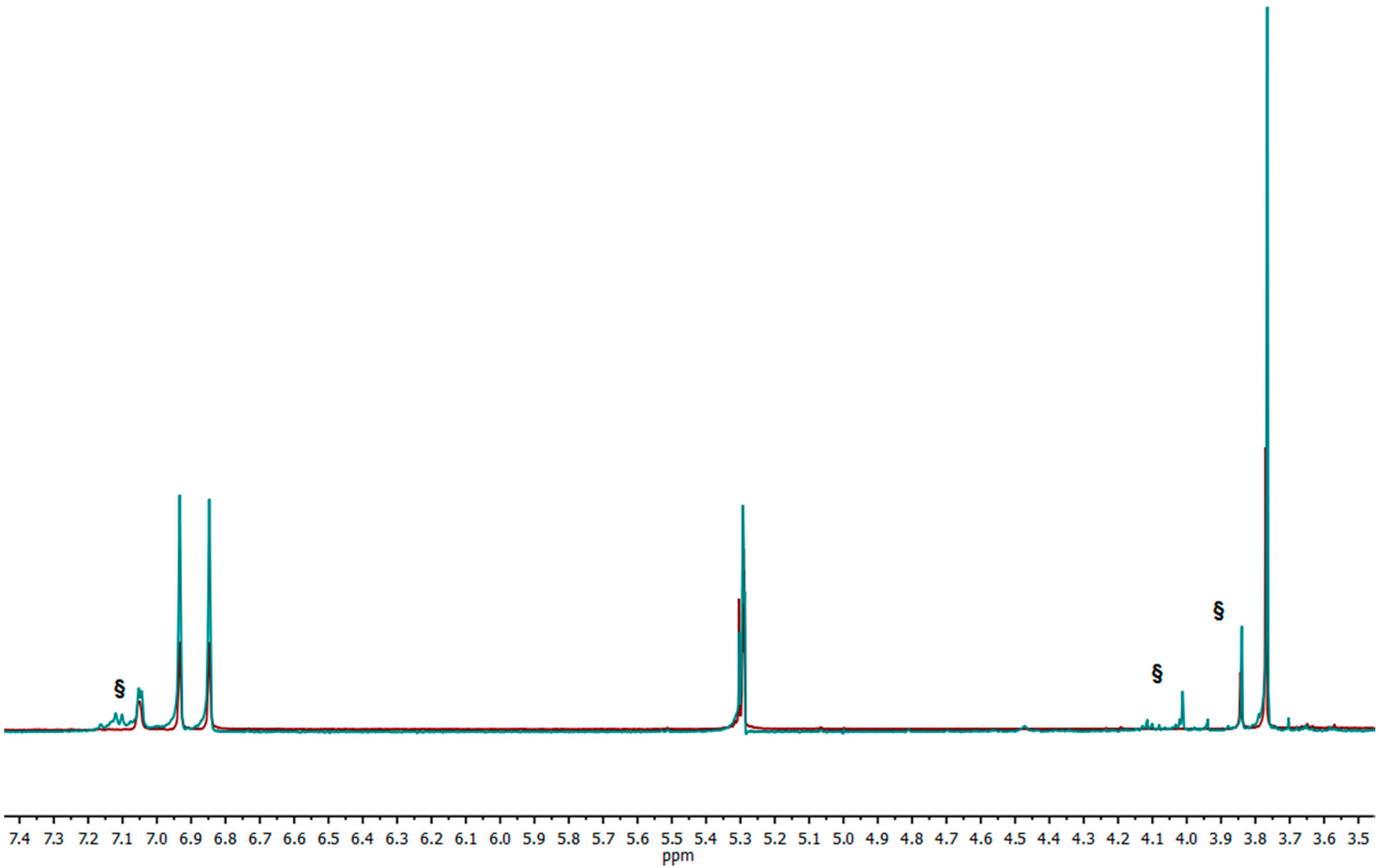

Figure S1. Superimposed ^{1}H-NMR spectra of **2á** (red line) and **2a** (blue line); § indicates small decomposition byproducts.

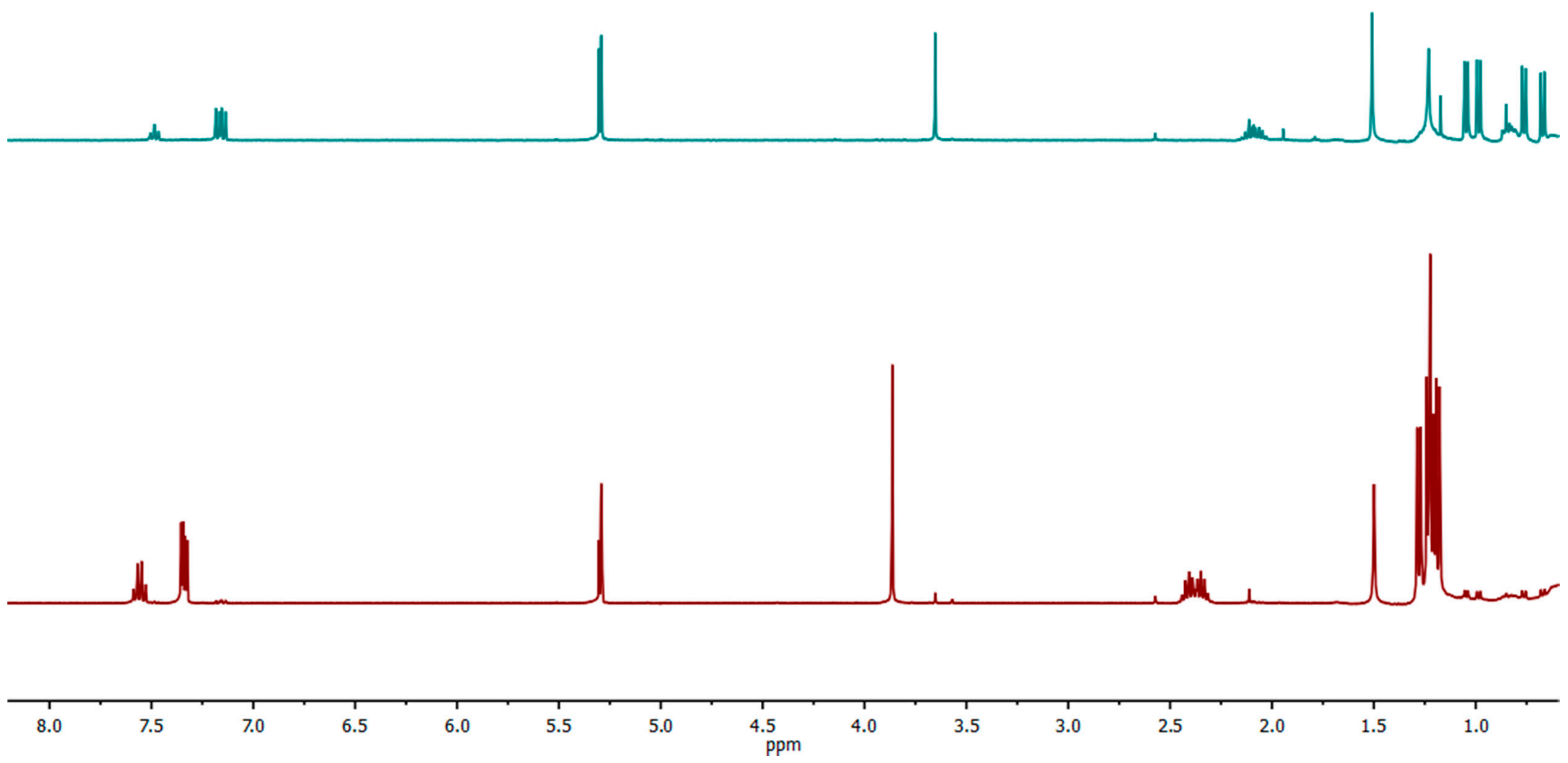

Figure S2. Stacked ^{1}H-NMR spectra of **1b** (red line) and **2b** (blue line).

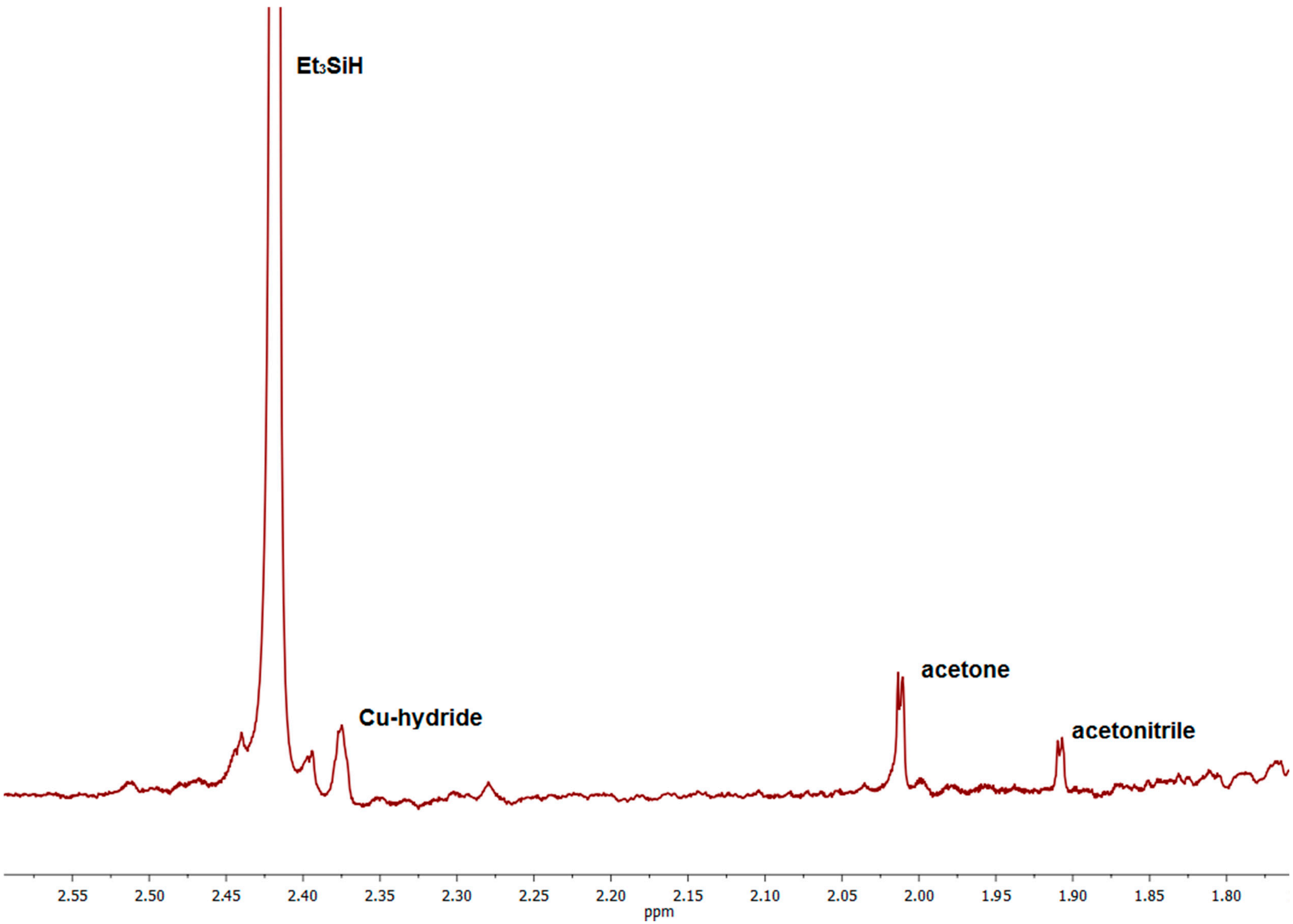

Figure S3. Potential hydride signal from the reaction of **1c** with Et_3SiH.
